# Função Ventricular Direita e Tolerância ao Exercício em Pacientes com Infarto Agudo do Miocárdio com Supradesnivelamento do Segmento ST

**DOI:** 10.36660/abc.20220799

**Published:** 2023-08-24

**Authors:** Denisse Guzman-Ramirez, Anival Trujillo-Garcia, Meredith Lopez-Rincon, Roxella Botello Lopez

**Affiliations:** 1 Hospital de Cardiologia UMAE Instituto Mexicano del Seguro Social Delegacion Nuevo Leon Departamento de Ecocardiografía Monterrey Nuevo Leon México Instituto Mexicano del Seguro Social Delegacion Nuevo Leon – Hospital de Cardiologia UMAE – Departamento de Ecocardiografía, Monterrey, Nuevo Leon – México

**Keywords:** Reabilitação cardíaca, Infarto do Miocárdio com Supradesnível do Segmento ST, Ecocardiografia, Função ventricular

## Abstract

**Fundamento:**

Pacientes com disfunção cardíaca apresentam limitações na realização de atividades físicas após a ocorrência de infarto do miocárdio com supradesnivelamento do segmento ST (IAMCSST). A função do ventrículo direito (VD) é determinante na melhora da capacidade funcional, sendo a reabilitação cardíaca (RC) essencial para essa coorte de pacientes.

**Objetivo:**

Avaliar a associação da função do VD com a tolerância ao exercício após um programa de RC em pacientes com IAMCSST.

**Métodos:**

Estudo de coorte retrospectivo em pacientes com IAMCSST, realizado de janeiro a dezembro de 2019. Os pacientes foram submetidos a uma avaliação ecocardiográfica da função do VD antes de um programa de RC de 16 sessões. Um teste de exercício cardiopulmonar (ECP) foi realizado antes e após o programa de RC. Analisamos se a função do VD, medida antes da RC, estava significativamente associada à tolerância ao exercício antes e depois do programa de RC e ao grau de melhora. Comorbidades e variáveis demográficas e anatômicas foram documentadas. Um valor de p < 0,05 foi considerado estatisticamente significativo.

**Resultados:**

No total, 109 pacientes foram incluídos. Destes, 3,7% apresentaram disfunção global do VD, 10,1% apresentaram disfunção radial do VD e 11% apresentaram disfunção longitudinal do VD. Observou-se associação entre a disfunção radial ou longitudinal do VD e a ausência de melhora da aptidão cardiorrespiratória (> 1 equivalente de pico de VO_2_) (p = 0,028, p = 0,008, respectivamente). Observou-se correlação significativa entre a disfunção longitudinal do VD com equivalentes de picos de VO_2_ (pVO_2_eq) iniciais (p = 0,046), pVO_2_eq final (p = 0,003) e diferença de pVO_2_eq (p = 0,009). Também foi identificada correlação entre a disfunção global do VD e pVO_2_eq inicial (p = 0,045), pVO_2_eq final (p = 0,012) e diferença de pVO_2_eq (p = 0,032).

**Conclusões:**

A disfunção do VD está associada a uma menor tolerância ao exercício; Os programas de RC podem ser estendidos ou modificados nesses pacientes.

## Introdução

A disfunção do ventrículo direito (VD) tem prevalência de 10% nos casos de infarto do miocárdio anteriores e de até 50% nos infartos inferiores.^1^ Ela é responsável por choque cardiogênico secundário à síndrome coronariana aguda em 5% dos casos.^2^ Em pacientes com infarto, a disfunção do VD é um importante fator prognóstico de mortalidade, arritmias ventriculares, complicações mecânicas, choque cardiogênico, trombose de stent e aumento da mortalidade em um ano e intra-hospitalar.^3 – 8^

Tajima et al. investigaram a função do VD, sua associação com a tolerância ao exercício e a eficácia da reabilitação cardíaca (RC) de fase II em pacientes com cardiopatia isquêmica. A disfunção do VD foi significativamente associada à 9% da redução da tolerância ao exercício antes da reabilitação. No entanto, a RC se mostrou eficaz nesses pacientes.^9^

Mahfouz et al. avaliaram a função do VD em pacientes com angina microvascular e sua relação com a tolerância ao exercício. Eles descobriram que um valor de tensão longitudinal do VD na parede livre ≤ −14,5% estava associado à redução da tolerância ao exercício.^10^

A RC para doença cardíaca coronária reduz mortalidade e hospitalizações e melhora a tolerância ao exercício e a qualidade de vida.^11^

A importância da melhora da capacidade física proporcionada pelo VD em outras patologias, como hipertensão pulmonar, regurgitação mitral e insuficiência cardíaca crônica, tem sido documentada; no entanto, sua importância nessa coorte de pacientes não foi estudada.^12 – 14^

Como nem todos os pacientes melhoram seus parâmetros de tolerância ao exercício em um programa de RC, há interesse em identificar fatores associados à resposta à tolerância ao exercício, programas ou preditores de resposta e não resposta.^15^ Poucas informações estão disponíveis sobre o papel da função do VD pós IAMCSST na tolerância ao exercício; sendo assim, mais estudos são necessários para definir se essas alterações impactam na classe funcional.

Portanto, o objetivo primário deste estudo foi avaliar a associação da função do VD com a tolerância ao exercício após um programa de RC em pacientes com IAMCSST tratados com intervenção coronária percutânea (ICP). Os objetivos secundários foram analisar os parâmetros da função do VD e frequência de disfunção ventricular antes da RC e comparar os equivalentes metabólicos do consumo de oxigênio (pVO_2_eq), representando a aptidão cardiorrespiratória e a classe funcional entre pacientes com e sem disfunção longitudinal, radial e global do VD.

## Métodos

Este estudo foi uma coorte retrospectiva analítica.

### População do estudo

Registros clínicos de RC de 109 pacientes adultos com IAMCSST, do sexo masculino e feminino, foram retrospectivamente revisados. Todos eles foram tratados com intervenção coronária percutânea no Hospital de Cardiologia UMAE n° 34 IMSS em Monterrey, Nuevo León, México, de 1° de janeiro de 2019 a 30 de janeiro de 2020. A ecocardiografia transtorácica foi realizada apenas antes da RC. Um teste de exercício cardiopulmonar (ECP) foi agendado antes e depois da RC. As comorbidades foram registradas quando diabetes mellitus, hipertensão, tabagismo e dislipidemia coexistiam. Os critérios de exclusão foram: ausência de dados ecocardiográficos antes da RC, pacientes com IAMCSST sem programa de RC, abandono da RC, ausência de achados do ECP antes e depois da RC, outros tipos de infarto do miocárdio (tipo 2, 4 e 5) e não-IAMCSST.

### Ecocardiografia

A função sistólica do VE e do VD foram avaliadas usando os equipamentos Vivid 9 e Vivid E95 da General Electric® para ecocardiografia transtorácica em repouso apenas antes da RC, medindo-se a fração de ejeção do ventrículo esquerdo (FEVE) e a fração de alteração da área do VD (FAC), a excursão sistólica do anel tricúspide (TAPSE) e velocidade sistólica do Doppler tissular (onda S’) do anel tricúspide. Com base nas diretrizes da *American Society of Echocardiography* , a disfunção do VE foi definida como FEVE < 50%. Para detectar a disfunção do VD com precisão, determinou-se sua caracterização por disfunção longitudinal do VD usando um ou mais dos seguintes critérios: TAPSE < 1,8 cm ou onda S’ < 9,5 cm/s. A disfunção radial do VD foi definida como FAC < 35% e a disfunção global do VD foi definida caso duas ou mais medições anteriores estivessem disponíveis.

### Teste cardiopulmonar de exercício

O ECP foi realizado em esteira da General Electric® T2100ST1 usando software CASE antes e após o programa de RC para estabelecer a aptidão cardiorrespiratória inicial e final. O programa de RC foi realizado em um ergômetro reclinado da Ergoline® GmbH, com aproximadamente 10 W/min de incrementos de carga a cada 5 minutos, até que os pacientes atingissem os critérios para finalização do teste (angina, alteração do segmento ST, dispneia, exaustão, hipotensão) ou completassem 40 minutos na esteira. No total, 15 a 20 sessões foram realizadas pelos pacientes, dependendo de sua situação específica. A medição contínua de pVO_2_eq foi realizada.

O Conselho de Ética em Pesquisa do Hospital de Cardiologia da UMAE Nº 34 IMSS aprovou o estudo, de acordo com o artigo 17 da seção de Aspectos Éticos da Pesquisa em Seres Humanos do Regulamento de Pesquisa da Lei Geral de Saúde do México. Este protocolo corresponde a um estudo de investigação sem risco, envolvendo revisão de registos clínicos, em que não foram identificados ou tratados aspectos sensíveis do comportamento do paciente.

### Análise estatística

Estatísticas descritivas foram calculadas com frequência e porcentagem para variáveis nominais. Variáveis contínuas, média, desvio padrão, mediana e intervalo interquartil foram relatados de acordo com a normalidade dos dados. Análises preliminares mostraram que a maioria dessas variáveis não apresentou distribuição normal, conforme avaliado pelo teste de Kolmogorov-Smirnov (p < 0,05). As exceções foram as variáveis idade e FAC, que apresentaram distribuição de curva normal (p = 0,131, p = 0,200, respectivamente). A análise inferencial das variáveis contínuas foi realizada de acordo com a curva de distribuição normal. A significância estatística foi considerada quando p < 0,05. A diferença na melhora cardiorrespiratória do pVO_2_eq antes e após a RC foi avaliada pelo teste de Wilcoxon. A associação entre a função (longitudinal e radial) do VD e a alteração da aptidão cardiorrespiratória (> 1 pVO_2_eq) no ECP após a RC foi avaliada com o teste exato de Fisher.

O tau-b de Kendall foi usado para avaliar a relação entre a função global e longitudinal do VD com pVO_2_eq inicial, pVO_2_eq final e a diferença no pVO_2_eq (p < 0,05). O teste Rho de Spearman foi realizado para determinar a correlação entre FEVE e Classe Funcional final e pVO_2_eq inicial (p < 0,05). A análise estatística foi realizada usando pacotes de software estatístico padrão (software SPSS 25.0; SPSS Inc, Chicago, IL, EUA e Office Excel; Washington, EUA).

## Resultados

Cento e nove (109) pacientes foram incluídos. As características demográficas e de IAMCSST são mostradas na Tabela 1 . A maioria dos pacientes eram homens, hipertensos e com dislipidemia. Eles apresentavam FEVE mediana de 49%, com uma CAF mediana de 45% da função radial do VD. Uma baixa porcentagem de pacientes apresentou disfunção radial, longitudinal e global do VD ( Tabela 1 ).

**Tabela 1:** Características basais do paciente, hemodinâmica, ecocardiograma e análise descritiva da RC

Demografia	N(%) / Med(IIQ) / M(DP)
Homens	90 (82,6)
Idade em anos	59 (9,9) **
Hipertensão	62 (56,9)
Tabagismo atual ou passado	47 (43,1)
Dislipidemia	74 (67,9)
Diabetes mellitus	58 (53,2)
**Hemodinâmica**	
Infarto do Miocárdio Anterior	60 (55)
Infarto do Miocárdio Inferior	41 (37,6)
Infarto do Miocárdio Lateral	8 (7,3)
Origem em Artéria Coronária Descendente	61 (56)
Origem em Artéria Coronária Direita	36 (33)
Origem em Artéria Coronária Circunflexa	12 (11)
**Ecocardiograma basal**	
FAC	45 (8,6) **
TAPSE	20,1 (4) *
Onda S'	12 (2) *
FEVE	49 (19) *
Disfunção longitudinal do VD	12 (11)
Disfunção radial do VD	11 (10,1)
Disfunção global do VD	4 (3,7)
**Reabilitação cardíaca**	
ECP basal (pVO_2_eq)	5,1 (2,9) *
Fim do ECP do programa de RC (pVO_2_eq)	7,3 (4,3) *
Diferença entre a linha de base e ECP inicial (pVO_2_eq)	2,1 (1,9) *

N: número de pacientes; %: porcentagem de pacientes; Med: mediano; IIQ: intervalo interquartil; M: nédia; DP: desvio padrão; FAC: fração de variação da área do ventrículo direito expressa em porcentagem; TAPSE: excursão sistólica do plano do anel tricúspide expressa em milímetros; Onda S': onda sistólica do ventrículo direito ao Doppler tissular expressa em centímetros por segundo; VD: ventrículo direito; FEVE: fração de ejeção do ventrículo esquerdo expressa em percentual; ECP: teste de esforço cardiopulmonar expresso como o pico de consumo de oxigênio em equivalentes metabólicos (pVO_2_eq); RC: programa de reabilitação cardíaca;

* Variáveis contínuas com distribuição assimétrica, mediana e amplitude interquartil são descritas.

** Variáveis contínuas com distribuição normal, média e desvio padrão são descritas.

O local mais comum de infarto foi o anterior, seguido dos inferiores. Com relação ao programa de RC, houve melhora considerável da pVO_2_eq. A variação mediana na aptidão cardiorrespiratória antes e após a RC foi de 2,1 pVO_2_eq, com diferença significativa (Z = −9,02 p = 0,001).

A maioria dos pacientes completou o programa de reabilitação em classe funcional I da New York Heart Association (NYHA), e a maioria dos pacientes apresentou FEVE superior a 40%. Figura Central .

**Figure:**
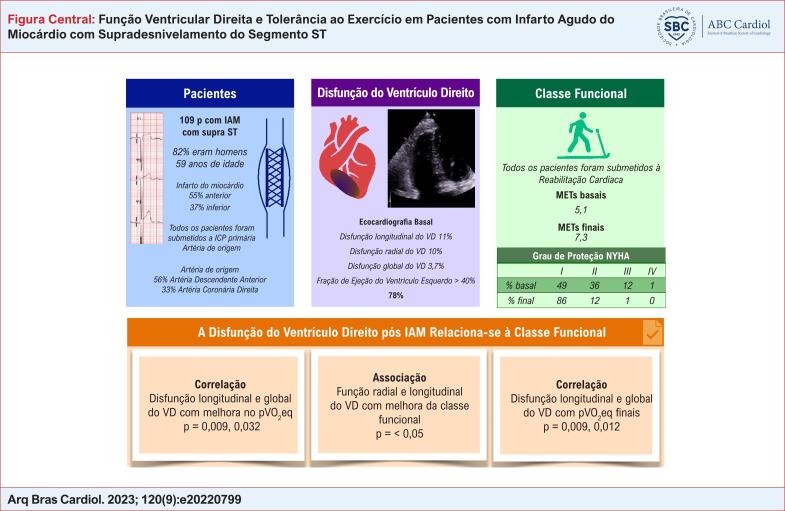
Disfunção do ventrículo direito e sua associação com a tolerância ao exercício. IAM: infarto agudo do miocárdio; ICP: intervenção coronária percutânea; VD: ventrículo direito; MET: equivalentes metabólicos do consumo de oxigênio; NYHA: New York Heart Association.

Observou-se diferença estatisticamente significativa ao comparar os pacientes com ou sem disfunção radial do VD e a presença de melhora na aptidão cardiorrespiratória (> 1 pVO_2_eq) (p = 0,028). A diferença observada ao comparar pacientes com ou sem disfunção longitudinal do VD e a melhora na aptidão cardiorrespiratória também foi significativa (p = 0,008) ( Tabela 2 ). Outra diferença significativa foi observada entre os pacientes com ou sem disfunção radial do VD e a presença de melhora da classe funcional NYHA (p = 0,031).

**Tabela 2:** Associação entre classe funcional e disfunção radial/longitudinal do VD

		Melhora da ACR (> 1 pVO_2_eq)		
		Sim	Não	N Total
	Normal	83	15	98
Função radial do VD	Disfunção	6	5	11
		Teste de Fisher p= 0,028		109
	Normal	83	14	97
Função longitudinal do VD	Disfunção	6	6	12
		Teste de Fisher p = 0,008		109

ACR: aptidão cardiorrespiratória; N: número de pacientes; VD: ventrículo direito; pVO_2_eq: equivalentes metabólicos do consumo de oxigênio.

**Figura 1:**
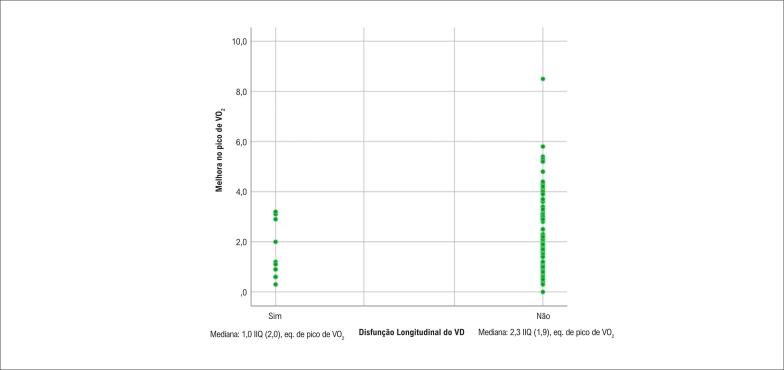
Correlação entre Disfunção Longitudinal do VD e Melhora no pVO_2_eq. IIQ: Intervalo interquartil; pico de eq. VO_2_ eq: pico de equivalentes de VO_2_ em pacientes com e sem disfunção longitudinal do VD.

A FAC do VD demonstrou uma correlação negativa significativa com a melhora na aptidão cardiorrespiratória (τb = −0,216 p = 0,010). A disfunção longitudinal e global do VD demonstrou correlações negativas significativas com pVO_2_eq inicial, pVO_2_eq final e melhora do pVO_2_eq. A FEVE teve correlação significativa com o pVO_2_eq inicial e com a classe funcional NYHA final ( Tabela 3 , Figuras 1 e 2 ).

**Figura 2:**
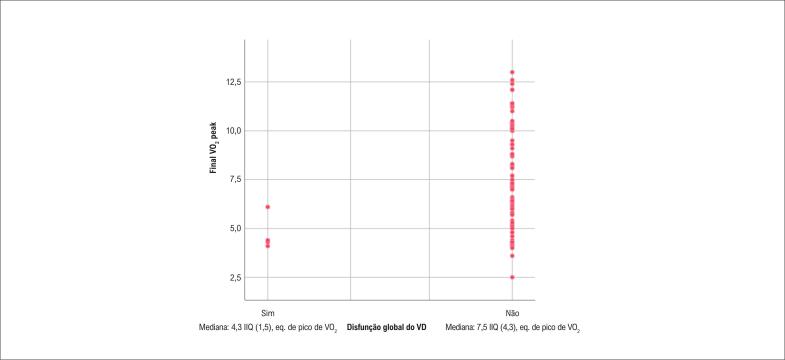
Correlação entre disfunção global do VD e pVO_2_eq final. IIQ: Intervalo interquartil; pico de eq. VO_2_ eq: pico de equivalentes de VO_2_ em pacientes com e sem disfunção global do VD.

**Tabela 3:** Parâmetros de função do VD versus parâmetros de aptidão cardiovascular

	Coeficiente de Correlação (valor de p)			
Variáveis	pVO_2_eq inicial	pVO_2_eq final	Melhoria do pVO_2_eq	Final CF NYHA
Disfunção longitudinal do VD	τb = 0,159 (0,046)	τb = 0,238 (0,003)	τb = 0,210 (0,009)	
Disfunção global do VD	τb = 0,160 (0,045)	τb = 0,201 (0,012)	τb = 0,171 (0,032)	
FEVE	ρ = 0,200 (0,036)			ρ = - 0.193 (0.044)

pVO_2_eq inicial: consumo de oxigênio em equivalentes metabólicos no ECP inicial; pVO_2_eq final: consumo de oxigênio em equivalentes metabólicos no ECP final; melhora no pVO_2_eq: diferença do consumo de oxigênio em equivalentes metabólicos entre o ECP inicial e final; CF NYHA: Classe Funcional da New York Heart Association; τb: correlação Tau-B de Kendall; FEVE: fração de ejeção do ventrículo esquerdo; ρ : Correlação Rho de Spearman.

## Discussão

Os principais achados do presente estudo são que as disfunções longitudinal e radial do VD estão associadas a pior aptidão cardiorrespiratória após a RC em pacientes com IAMCSST tratados com ICP primária. Em estudos anteriores, Tajima et al. documentaram que a função global do VD determina significativamente a capacidade de exercício na cardiopatia isquêmica antes da RC. No entanto, os pacientes incluídos eram heterogêneos, com doença arterial coronariana multiarterial tratada com ICP ou cirurgia de revascularização do miocárdio.^9^ Em nosso estudo, a ecocardiografia basal também evidenciou tal associação.

A disfunção radial do VD, avaliada como FAC, foi associada à ausência de melhora da aptidão cardiorrespiratória (> 1 pVO_2_eq), sugerindo que as fibras radiais do VD estão envolvidas na capacidade funcional desses pacientes. A observação de que pVO_2_eq inicial, final e melhorado apresentaram correlações com disfunção longitudinal e global do VD é consistente com outros estudos, que mostraram a mesma relação entre disfunção do VD e baixa tolerância ao exercício, fato relacionado ao desacoplamento ventrículo-arterial pulmonar.^14 , 16^

Legris et al. observaram que, em pacientes com insuficiência cardíaca, a disfunção do VD avaliada pela FAC e pelo índice de desempenho miocárdico estava associada a estudos de tolerância ao exercício; no entanto, a função longitudinal não mostrou essa relação. Esses achados indicam que o método apresente condições dependentes de pré-carga.^16^

Muitos estudos compararam a função do VD em pacientes com cardiopatia isquêmica submetidos à RC, usando medição ecocardiográfica de TAPSE e velocidade de S'.^9 , 14 , 15^ Em pacientes tratados com enxertos de revascularização do miocárdio, uma redução desses parâmetros para, pelo menos, metade dos valores normais parece ser uma característica típica da cirurgia cardíaca não complicada. Essa redução tem sido relacionada à abertura do pericárdio, alterações durante a CEC e a esternotomia. Ainda assim, ela não indica, necessariamente, um comprometimento da função do VD, e essas alterações persistem por 12 a 24 meses.^17^ Por esse motivo, apenas pacientes em tratamento percutâneo foram incluídos.

Apenas 33% dos pacientes apresentavam doença arterial coronariana direita; esses resultados sugerem que a disfunção do VD não está apenas relacionada à doença multivascular ou à doença da artéria coronária direita, mas, uma vez presente, é também uma entidade independente com prognóstico ruim. Estudos anteriores relataram tais achados em pacientes com infarto agudo do miocárdio e choque cardiogênico com e sem doença da artéria coronária descendente anterior esquerda.^8 , 18^

Os resultados deste estudo sugerem que, embora haja melhora na classe funcional e no consumo de VO_2_ nesses pacientes, a disfunção do VD está associada a uma menor resposta à tolerância ao exercício em programas de RC. Eles também mostram que a disfunção do VD persiste após a RC, com possíveis repercussões futuras.

No entanto, em nosso estudo, todos os pacientes apresentaram melhora da classe funcional e VO^2^, o que se refletiu na diferença de pVO_2_eq, com máximo de 3,8. Pacientes com disfunção do VD, tanto longitudinal quanto radial, apresentaram melhora da aptidão cardiorrespiratória (> 1 pVO_2_eq) ao final da RC. Provavelmente, esses pacientes adquiriram os benefícios previamente reconhecidos em outros estudos, nos quais a melhora em um VO^2^ equivalente diminuiu a mortalidade em 25%.^19^ Esses achados sugerem que a disfunção do VD diminui a capacidade de resposta à atividade física; no entanto, isso não limita totalmente os benefícios da RC. Além disso, essa população pode se beneficiar ainda mais de um programa prolongado ou de diferentes rotinas de exercícios para melhora da classe funcional. Esses programas afetam positivamente os pacientes selecionados, mesmo com as limitações mencionadas. Essa situação foi abordada no estudo de Ohara et al.; em que pacientes com baixa TAPSE, submetidos a RC por seis meses, apresentaram melhora da classe funcional, reduziram os níveis de peptídeo natriurético tipo B, reduziram a pós-carga do VD e melhoraram a função sistólica do ventrículo esquerdo, embora não se tenha observado alterações na TAPSE em um estudo ecocardiográfico final. Os resultados sugerem que uma estratégia de RC mais estendida poderia melhorar a aptidão cardiorrespiratória desses pacientes.^14^

Conforme relatado anteriormente, a FEVE correlacionou-se com a classe funcional e pVO_2_eq inicial. A disfunção do VE está relacionada à intolerância ao exercício devido à reserva sistólica prejudicada, o que prejudica ainda mais a reserva do volume sistólico durante o estresse.^20^

O benefício da RC se reflete no fato de que 86% dos pacientes concluíram o programa de RC em classe funcional I, com melhora média de 2,4 pVO_2_eq ± 1,4; apenas 12,5% dos pacientes concluíram a reabilitação na classe funcional II e 0,9% na classe III. Esse dado foi estudado anteriormente em pacientes com insuficiência cardíaca, e resultados semelhantes foram encontrados com TAPSE.^14^ Sendo assim, a disfunção do VD está associada a uma menor capacidade de tolerância ao exercício; no entanto, isso não diminui os benefícios dos programas de reabilitação para esses pacientes.

As estratégias de reabilitação mais adequadas devem ser estabelecidas para esses pacientes. Os parâmetros da função do VD podem ajudar a caracterizar a menor tolerância ao exercício e individualizar o programa para que se possa obter os melhores resultados a curto prazo e provavelmente a longo prazo.

Uma das limitações deste estudo é a baixa frequência de pacientes com disfunção longitudinal, radial e global do VD. Outra limitação é que a duração total de cada sessão de reabilitação não foi considerada, embora o tempo máximo de cada sessão fosse de 40 minutos. O consumo direto de oxigênio do paciente não foi medido, nem a sessão foi suspensa por qualquer motivo, pois apenas pacientes com programa de RC completo foram incluídos; a sub-representação das mulheres foi outro fator de viés.

Mais estudos, que incluam o acompanhamento desses pacientes, além da avaliação do consumo de oxigênio em correlação com a tolerância ao exercício e a função do VD são necessários, além de estudos que avaliem os resultados a longo prazo.

## Conclusões

A disfunção do VD em pacientes com cardiopatia isquêmica com IAMCSST prévio e intervenção coronária percutânea primária está associada e se correlaciona com tolerância ao exercício e classe funcional reduzidas antes da RC. Após a RC, houve melhora do pVO_2_eq.
