# Supplementary material for: Função Ventricular Direita e Tolerância ao Exercício em Pacientes com Infarto Agudo do Miocárdio com Supradesnivelamento do Segmento ST
Source: Arq Bras Cardiol. 2023 Aug 24;120(9):e20220799. [Article in Portuguese] doi: 10.36660/abc.20220799 (PMC10519353; doi:10.36660/abc.20220799)
Supplement: Translation

# Translation: Right Ventricular Function and Exercise Tolerance in Patients with ST-Elevation Myocardial Infarction

**Authors:** Denisse Guzman-Ramirez, Anival Trujillo-Garcia, Meredith Lopez-Rincon, Roxella Botello Lopez

**Affiliations:** 1 Monterrey Nuevo Leon Mexico Instituto Mexicano del Seguro Social Delegacion Nuevo Leon – Hospital de Cardiologia UMAE – Departamento de Ecocardiografía, Monterrey, Nuevo Leon – Mexico

**Keywords:** Cardiac Rehabilitation, ST Elevation Myocardial Infarction, Echocardiography, Ventricular Function

## Abstract

**Background:**

Following ST-segment elevation myocardial infarction (STEMI), patients with cardiac dysfunction have limitations in performing physical activity. Right ventricular (RV) function is a determinant in improving functional capacity, and cardiac rehabilitation (CR) is essential for this patient cohort.

**Objective:**

To evaluate the association of RV function with exercise tolerance after a CR program in patients with STEMI.

**Methods:**

Retrospective cohort study in patients with STEMI from January to December 2019. They underwent an echocardiographic assessment of RV function before a 16-session CR program. A cardiopulmonary exercise (CPX) testing was performed before and after the CR program. We analyzed whether RV function measured before CR was significantly associated with exercise tolerance before and after the CR program and the degree of improvement. Comorbidity, demographic and anatomic variables were documented. A p-value < 0.05 was considered statistically significant.

**Results:**

A total of 109 patients were included. Of all, 3.7% had global RV dysfunction, 10.1% had radial RV dysfunction, and 11% had longitudinal RV dysfunction. An association between radial or longitudinal RV dysfunction and the absence of improvement in cardiorespiratory fitness (> 1 peak VO_2_equivalents) was observed (p = 0.028, p = 0.008, respectively). A significant correlation was observed between longitudinal RV dysfunction with initial peak VO_2_equivalents (pVO_2_eq) (p = 0.046), final pVO_2_eq (p = 0.003), and difference in pVO_2_eq (p = 0.009). A correlation was also identified between global RV dysfunction and the initial pVO_2_eq (p = 0.045), final pVO_2_eq (p = 0.012), and difference in pVO_2_eq (p = 0.032).

**Conclusions:**

RV dysfunction is associated with a lower capacity to exercise; CR programs can be extended or modified in these patients.

## Introduction

Right ventricle (RV) dysfunction has a prevalence of 10% in anterior myocardial infarctions and up to 50% in inferior myocardial infarctions.^1^ It is responsible for cardiogenic shock secondary to an acute coronary syndrome in 5% of cases.^2^ In patients with myocardial infarction, RV dysfunction is an important prognostic factor of mortality, ventricular arrhythmias, mechanical complications, cardiogenic shock, stent thrombosis, and increased in-hospital and one-year mortality.^3 – 8^

Tajima et al. investigated RV function, its association with exercise tolerance, and the efficacy of phase II cardiac rehabilitation (CR) in patients with ischemic heart disease. RV dysfunction was significantly associated with reduced exercise tolerance by 9% before rehabilitation. However, CR was effective in these patients.^9^

Mahfouz et al. evaluated RV function in patients with microvascular angina and its relationship with exercise tolerance. They found that a value of ≤ −14.5% of free wall longitudinal RV strain was associated with reduced exercise capacity.^10^

CR for coronary heart disease reduces mortality and hospitalizations and improves exercise capacity and quality of life.^11^

The importance of improving the physical capacity that the RV provides in other pathologies, such as pulmonary hypertension, mitral regurgitation, and chronic heart failure, has been documented; however, its importance in this patient cohort has not been studied.^12 – 14^

Since not all patients improve their exercise tolerance parameters in a CR program, there is interest in identifying factors associated with response to exercise tolerance, programs, or predictors of response and non-response.^15^ There is scarce information about the role of RV function after STEMI in exercise tolerance; thus, whether these alterations impact functional class needs to be better defined.

Therefore, the primary objective of this study was to evaluate the association of RV function with exercise tolerance after a CR program in patients with STEMI treated with percutaneous coronary intervention. Secondary objectives were to analyze RV function parameters and ventricular dysfunction frequency before CR and compare metabolic equivalents of oxygen consumption (pVO_2_eq), representing cardiorespiratory fitness and functional class between patients with and without longitudinal, radial, and global RV dysfunction.

## Methods

This study was an analytical retrospective cohort.

### Study population

We retrospectively reviewed CR clinical records of 109 adult male and female patients with STEMI. All of them were treated with percutaneous coronary intervention in the UMAE Hospital of Cardiology No. 34 IMSS in Monterrey, Nuevo León, Mexico, from January 1, 2019 to January 30, 2020. Transthoracic echocardiography was performed only before CR. A cardiopulmonary exercise (CPX) testing was scheduled before and after CR. Comorbidities were recorded when diabetes mellitus, hypertension, smoking, and dyslipidemia coexisted. Exclusion criteria were: absence of echocardiographic data before CR, patients with STEMI but without a CR program, the dropout from CR, absence of CPX findings before and after CR, other types of myocardial infarction (type 2, 4, and 5), and non-STEMI.

### Echocardiography

Systolic LV function and RV function were evaluated using Vivid 9 and Vivid E95 General Electric® equipment for resting transthoracic echocardiography only before CR, measuring left ventricle ejection fraction (LVEF) and RV fractional area change (RVFAC), tricuspid annulus systolic excursion (TAPSE) and tissue Doppler systolic velocity (S’ wave) of the tricuspid annulus. Based on the American Society of Echocardiography guidelines, LV dysfunction was defined as LVEF < 50%. To accurately detect RV dysfunction, we determined whether it is characterized by longitudinal RV dysfunction using one or more of the following criteria: TAPSE < 1.8 cm or S' wave < 9.5 cm/s. Radial RV dysfunction was defined as RVFAC < 35% and global RV dysfunction was defined if two or more previous measurements were available.

### Cardiopulmonary exercise testing

CPX was performed on a General Electric® T2100ST1 treadmill with CASE software before and after the CR program to establish initial and final cardiorespiratory fitness. The CR program was performed on a recumbent Ergoline® GmbH ergometer with approximately 10 W/min workload increments every 5 minutes until the subjects achieved the criteria for test termination (angina, ST segment change, dyspnea, exhaustion, hypotension) or completed 40 minutes on the treadmill. In total, 15 to 20 sessions were completed by patients, depending on their specific situation. Continuous measurement of pVO_2_eq was carried out.

The Research Ethics Board of the UMAE Hospital of Cardiology No. 34 IMSS approved the study, according to article 17 of the section on Ethical Aspects of Human-Subject Research of the General Health Law Research Regulations in Mexico. This protocol corresponds to a research study without risk, involving reviewing clinical records, in which no sensitive aspects of a patient's behavior were identified or treated.

### Statistical analysis

Descriptive statistics were calculated with frequency and percentage for nominal variables. For continuous variables, mean, standard deviation, median, and interquartile range were reported according to data normality. Preliminary analyses showed that most of these variables were not normally distributed, as assessed by the Kolmogorov-Smirnov test (p < 0.05). The exceptions were the variables age and RVFAC, which had a normal curve distribution (p = 0.131, p = 0.200, respectively). Inferential analysis of continuous variables was performed according to the normal distribution curve. Statistical significance was considered when p < 0.05. The difference in cardiorespiratory improvement in pVO_2_eq before and after CR was assessed by the Wilcoxon test. The association between (longitudinal and radial) RV function and change in cardiorespiratory fitness (> 1 pVO_2_eq) in CPX after CR was assessed with Fisher's exact test.

Kendall's tau-b was used to assess the relationship between global and longitudinal RV function with initial pVO_2_eq, final pVO_2_eq, and the difference in pVO_2_eq (p < 0.05). Spearman's Rho test was conducted to determine the correlation between LVEF and final Functional Class, and initial pVO_2_eq (p < 0.05). Statistical analysis was performed using standard statistical software packages (SPSS software 25.0; SPSS Inc, Chicago, IL, USA, and Office Excel; Washington, USA).

## Results

We included 109 patients. Demographics and STEMI characteristics are shown in Table 1 . Most of the patients were men, hypertensive, and with dyslipidemia. They had a median LVEF of 49%, with a median CAF of 45% of radial RV function. A low percentage of patients had radial, longitudinal, and global RV dysfunction ( Table 1 ).

**Table 1:** Patient baseline characteristics, hemodynamics, echocardiogram and CR descriptive analysis

Demographics	N(%) / Med(IQR) / M(SD)
Men	90 (82.6)
Age in years	59 (9.9) **
Hypertension	62 (56.9)
Current or past smoking	47 (43.1)
Dyslipidemia	74 (67.9)
Diabetes mellitus	58 (53.2)
**Hemodynamics**	
Anterior Myocardial Infarction	60 (55)
Inferior Myocardial Infarction	41 (37.6)
Lateral Myocardial Infarction	8 (7.3)
Culprit Anterior Descending Coronary Artery	61 (56)
Culprit Right Coronary Artery	36 (33)
Culprit Circumflex Coronary Artery	12 (11)
**Basal Echocardiogram**	
FAC	45 (8.6) **
TAPSE	20.1 (4) *
S' wave	12 (2) *
LVEF	49 (19) *
Longitudinal RV dysfunction	12 (11)
Radial RV dysfunction	11 (10.1)
Global RV dysfunction	4 (3.7)
**Cardiac Rehabilitation**	
Baseline CPX (pVO_2_eq)	5.1 (2.9) *
End of CR program CPX (pVO_2_eq)	7.3 (4.3) *
Difference between baseline and final CPX (pVO_2_eq)	2.1 (1.9) *

N: number of patients; %: percentage of patients; Med: median; IQR: interquartile range; M: Mean; SD: Standard deviation; FAC: right ventricle fractional area change expressed in percentage; TAPSE: tricuspid annulus plane systolic excursion expressed in millimeters; S' wave: tissue Doppler right ventricle systolic wave expressed in centimeters per second; RV: right ventricle; LVEF: left ventricle ejection fraction expressed in percentage; CPX: cardiopulmonary stress test expressed in peak oxygen consumption in metabolic equivalents (pVO_2_eq); CR: cardiac rehabilitation program.

* Continuous variables with asymmetric distribution, median and interquartile range are described.

** Continuous variables with normal distribution, mean and standard deviation are described.

The most common site of infarction was anterior, followed by inferior ones. Regarding the CR program, there was a considerable improvement in pVO_2_eq. The median change in cardiorespiratory fitness before and after CR was 2.1 pVO_2_eq, the difference being significant (Z = −9.02 p = 0.001).

Most patients completed the rehabilitation program in New York Heart Association (NYHA) functional class I, and most patients had an LVEF of more than 40%. Central Illustration .

**Figure:** Right ventricle dysfunction and its association with exercise tolerance. AMI: acute myocardial infarction; PCI: percutaneous coronary intervention; RV: right ventricle; MET: metabolic equivalents of oxygen consumption; NYHA: New York Heart Association.

A statistically significant difference was observed comparing patients with or without radial RV dysfunction and the presence of improvement in cardiorespiratory fitness (> 1 pVO_2_eq) (p = 0.028). The difference between comparing patients with or without longitudinal RV dysfunction and improvement in cardiorespiratory fitness was also significant (p = 0.008) ( Table 2 ). Another significant difference was observed between patients with or without radial RV dysfunction and the presence of improved NYHA functional class (p = 0.031).

**Table 2:** Association between functional class and radial/longitudinal RV dysfunction

		CF Improvement (> 1 pVO_2_eq)		
		Yes	No	N Total
	Normal	83	15	98
Radial RV Function	Dysfunction	6	5	11
		Fisher Test p = 0.028		109
	Normal	83	14	97
Longitudinal RV Function	Dysfunction	6	6	12
		Fisher Test p = 0.008		109

CF: cardiorespiratory fitness; N: number of patients; RV: right ventricle; pVO_2_eq: metabolic equivalents of oxygen consumption.

RV FAC had a significant negative correlation with improvement in cardiorespiratory fitness (τb = −0.216 p = 0.010). Longitudinal and global RV dysfunction had significant negative correlations with initial pVO_2_eq, final pVO_2_eq, and improvement in pVO_2_eq. LVEF had a significant correlation with initial pVO_2_eq and with final NYHA functional class ( Table 3 , Figures 1 and 2 ).

**Figure 1:** Correlation between Longitudinal RV Dysfunction and Improvement in pVO_2_eq. IQR: interquartile range; peak VO_2_eq: peak VO_2_equivalents of patients with and without RV longitudinal dysfunction.

**Figure 2:** Correlation between RV Global Dysfunction and Final pVO_2_eq. IQR: interquartile range; peak VO_2_eq: peak VO_2_equivalents of patients with and without RV global dysfunction.

**Table 3:** RV function parameters versus cardiovascular fitness parameters

	Correlation Coefficient (p-value)			
Variables	Initial pVO_2_eq	Final pVO_2_eq	Improvement in pVO_2_eq	Final NYHA FC
Longitudinal RV dysfunction	τb = 0.159 (0.046)	τb = 0.238 (0.003)	τb = 0.210 (0.009)	
Global RV dysfunction	τb = 0.160 (0.045)	τb = 0.201 (0.012)	τb = 0.171 (0.032)	
LVEF	ρ = 0.200 (0.036)			ρ = - 0.193 (0.044)

Initial pVO_2_eq: oxygen consumption in metabolic equivalents in initial CPX; final pVO_2_eq: oxygen consumption in metabolic equivalents in the final CPX; improvement in pVO_2_eq: difference of oxygen consumption in metabolic equivalents between initial and final CPX; NYHA FC: New York Heart Association Functional Class; τb: Kendall's Tau-B correlation; LVEF: left ventricle ejection fraction; ρ : Spearman's Rho correlation.

## Discussion

The main findings of the present study are that longitudinal and radial RV dysfunction are associated with worse cardiorespiratory fitness after CR in patients with STEMI treated with primary PCI. In previous studies, Tajima et al. documented that global RV function significantly determines exercise capacity in ischemic heart disease before CR. Nevertheless, the patients included were heterogeneous with multivessel coronary artery disease treated with PCI or coronary artery bypass surgery.^9^ In our study, basal echocardiography also showed this association.

We found that radial RV dysfunction, evaluated as FAC, was associated with a lack of improvement in cardiorespiratory fitness (> 1 pVO_2_eq); this suggests that radial RV fibers are involved in the functional capacity of these patients. The observation that initial, final, and improvement of pVO_2_eq showed correlations with longitudinal and global RV dysfunction is consistent with other studies that showed this relationship between RV dysfunction and poor exercise tolerance, a fact related to ventricle-pulmonary artery uncoupling.^14 , 16^

Legris et al. observed that in patients with heart failure, RV dysfunction assessed using FAC and myocardial performance index was associated with exercise tolerance studies; however, longitudinal function did not show this relationship. These findings reflect the preload-dependent conditions of this method.^16^

Many studies have compared RV function in patients with ischemic heart disease undergoing CR, using echocardiographic measurement of TAPSE and S' velocity.^9 , 14 , 15^ In patients treated with coronary artery bypass grafts, a reduction in these parameters to at least half of normal values seems to be a typical feature of uncomplicated cardiac surgery. This reduction has been related to the opening of the pericardium, changes during CPB, and sternotomy. Still, it does not necessarily indicate impairment of RV function, and these changes persist for 12 to 24 months.^17^ For this reason, we only enrolled patients undergoing percutaneous treatment.

Only 33% of the patients had right coronary artery disease; these results suggest that RV dysfunction is not only related to multi-vascular disease or right coronary artery disease but is also an independent entity with a poor prognosis once it is present. Previous studies reported such findings in patients with acute myocardial infarction and cardiogenic shock with and without left anterior descendant coronary artery disease.^8 , 18^

The results of this study suggest that although there is an improvement in functional class and VO_2_consumption in these patients, RV dysfunction is associated with a lower response to exercise tolerance in CR programs. They also show that RV dysfunction persists after CR and possibly has future repercussions.

Nevertheless, in our study, all patients improved in functional class and VO_2_; this was reflected in the difference in pVO_2_eq, with a maximum of 3.8. Patients with RV dysfunction, both longitudinal and radial, showed improved cardiorespiratory fitness (> 1 pVO_2_eq) at the end of CR. Therefore, these patients probably acquired the benefits previously recognized in other studies where improvement by one VO_2_equivalent decreased mortality by 25%.^19^ These findings suggest that RV dysfunction decreases the capacity to respond to physical activity; however, this does not entirely limit the benefits of CR. Moreover, this population may benefit more from an extended program or different exercise routines to improve their functional class. These programs positively affect selected patients even with the limitations mentioned. This situation was addressed in the study by Ohara et al.; they found that patients with low TAPSE who underwent CR for six months improved their functional class, reduced B-type natriuretic peptide levels, reduced afterload of the right ventricle and improved left ventricle systolic function even though the TAPSE did not change in a final echocardiographic study. The results suggest that a more extended CR strategy could improve the cardiorespiratory fitness of these patients.^14^

As reported previously, LVEF correlated with functional class and initial pVO_2_eq. LV dysfunction is related to exercise intolerance because of the impaired systolic reserve, which further impairs stroke volume reserve during stress.^20^

The benefit of CR is reflected in the fact that 86% of the patients finished the CR program in functional class I, with an average improvement of 2.4 pVO_2_eq ± 1.4; only 12.5% ended up in functional class II and 0.9% in class III. This data was studied previously in patients with heart failure, and similar results were found with TAPSE.^14^ Therefore, RV dysfunction is associated with a lower exercise tolerance capacity; nevertheless, this does not reduce the benefits of rehabilitation programs for these patients.

The most appropriate rehabilitation strategies should be established for these patients. RV function parameters can help to characterize lower exercise tolerance and individualize the program to obtain the best short-term and probably long-term results.

Among the limitations of this study is the low frequency of patients with longitudinal, radial, and global RV dysfunction. Another limitation is that the total duration of each rehabilitation session was not considered, although the maximum time for each session was 40 minutes. The patient's direct oxygen consumption was not measured, nor was the session suspended for any reason since only patients with a complete CR program were included; the underrepresentation of women was another bias factor.

Further studies are needed in which the follow-up of these patients is included in addition to the evaluation of oxygen consumption in correlation with exercise tolerance and RV function, as well as assessing long-term outcomes.

## Conclusions

RV dysfunction in patients with ischemic heart disease with previous STEMI and primary percutaneous coronary intervention is associated and correlates with lower exercise tolerance and functional class before CR. After CR, the final pVO_2_eq was improved.
